# The sugar and energy in non-carbonated sugar-sweetened beverages: a cross-sectional study

**DOI:** 10.1186/s12889-019-7486-6

**Published:** 2019-08-20

**Authors:** Chuyao Jin, Lizi Lin, Chenxiong Li, Yuanzhou Peng, Graham A. MacGregor, Fengjun He, Haijun Wang

**Affiliations:** 1 Department of Maternal and Child Health, School of Public Health, Peking University, No. 38, Xueyuan Rd, Haidian District, Beijing, 100191 People’s Republic of China; 2 Wolfson Institute of Preventive Medicine, Barts and The London School of Medicine and Dentistry, Queen Mary University of London, London, UK

**Keywords:** Sugar, Energy, Sugar-sweetened beverages, Serving size

## Abstract

**Background:**

The consumption of non-carbonated sugar-sweetened beverages (NCSSBs) has many adverse health effects. However, the sugar and energy content in NCSSBs sold in China remain unknown. We aimed to investigate the sugar and energy content of NCSSBs in China and how these contents were labelled.

**Methods:**

A cross-sectional survey was conducted in 15 supermarkets in Haidian District, Beijing from July to October 2017. The product packaging and nutrient information panels of NCSSBs were recorded to obtain type of products (local/imported), serving size, nutrient contents of carbohydrate, sugar and energy. For those NCSSBs without sugar content information, we used carbohydrate content as a replacement.

**Results:**

A total of 463 NCSSBs met the inclusion criteria and were included in our analysis. The median of sugar content and energy content was 9.6 [interquartile range (IQR): 7.1–11.3] g/100 ml and 176 (IQR: 121–201) kJ/100 ml. The median of sugar contents in juice drinks, tea-based beverages, sports drinks and energy drinks were 10.4, 8.5, 5.0 and 7.4 g/100 ml. Imported products had higher sugar and energy content than local products. There were 95.2% products of NCSSBs receiving a ‘red’(high) label for sugars per portion according to the UK criteria, and 81.6% products exceeding the daily free sugar intake recommendation from the World Health Organization (25 g). There were 82 (17.7%) products with sugar content on the nutrition labels and 60.2% of them were imported products.

**Conclusions:**

NCSSBs had high sugar and energy content, and few of them provided sugar content information on their nutrition labels especially in local products. Measures including developing better regulation of labelling, reducing sugar content and restricting the serving size are needed for reducing sugar intakes in China.

**Electronic supplementary material:**

The online version of this article (10.1186/s12889-019-7486-6) contains supplementary material, which is available to authorized users.

## Background

Free sugar in sugar-sweetened beverages (SSBs) received considerable attention from public and emerged as a popular health issue. The term ‘free sugar’ refers to monosaccharides and disaccharides added to foods and beverages by the manufacturer, cook or consumer, and sugars naturally present in honey, syrups, fruit juices and fruit juice concentrates [1]. With the rapid urbanization and westernization of the diet pattern in China, the annual per-capita consumption of SSBs has more than tripled in the last decade and a half (16.9 kg in 2003, 59.7 kg in 2017) [2]. Large amount of free sugars in SSBs with high calories content and nonnutritive value [3] are associated with increased risk of obesity, type 2 diabetes, hypertension, dental caries and other non-communicable diseases [4, 5].

The most well-known type of SSBs are carbonated products such as soda. With the increasing studies and rapid spread of mass media, the public has improved the awareness of the high sugar content and hazard of carbonated SSBs (CSSBs) [6–8]. As a result, some people preferred non-carbonated SSBs (NCSSBs) as potential alternatives to CSSBs with the perception that they are healthier [9]. NCSSBs are SSBs without carbon dioxide including juice drinks, tea-based beverages, sports drinks, and energy drinks [10]. The fruit mostly serves as a flavoring in juice drinks with higher water content, while only fruit juice (100% juice) showed preventative effects on hypertension, inflammation and cancer [11]. The polyphenol in tea is an antioxidant that helps protect cellular damage [12]. Sports drinks help athletes to maintain optimal performance and replace electrolytes and fluids lost during vigorous physical activities [13]. While in energy drinks, high caffeine content provides effect on reducing feelings of tiredness and enhancing mental alertness [14]. However, the harmful effects of sugars in these NCSSBs may overweigh these seemingly attractive functions. Studies have proved that high consumption of solid fruit is associated with lower risk of diabetes, while high juice drink intake didn’t show similar effects [15]. Likewise, the healthful effects of hot tea (soaking the dried tea leaves in hot water) were subdued when tea was consumed in ready-to-drink (RTD) cold tea form, mainly because of its high sugar content [16] and lower antioxidant ingredients [17]. Furthermore, the high sugar and caffeine contents in sports and energy drinks have been associated with increased risk of obesity, dental erosion, type 2 diabetes, palpitations, hypertension, and other diseases [18, 19]. Meanwhile, labelling carbohydrate content information is mandatory while labelling sugar content information is voluntary based on the released Regulation for Food Nutrition Labelling [20]. Therefore, consumers in China might show misconception or neglect of the sugar content in NCSSBs [21].

Importantly, the sugar contents of the same NCSSBs were different across different countries [22]. Although high sugar contents of these products have been found in developed countries [7, 8, 23, 24], no study has reported the sugar and energy content in NCSSBs in China. Considering China has the world’s second largest market of SSBs with the rapidly expanding market of NCSSBs [2], with the growing interest in a policy intervention to reduce the consumption of SSBs at a population level, research to understand the sugar content and energy content in NCSSBs sold in China are urgently needed. Therefore, we conducted a cross-sectional study in 15 supermarkets in Haidian District, Beijing from July to October 2017, aiming to investigate the sugar and energy content of NCSSBs in China and how these contents were labelled. We hypothesize that the sugar and energy content in NCSSBs in China are high and most NCSSBs lack sugar content on the nutrition labels.

## Methods

### Selection of supermarket

The information of the total number of markets and their addresses in Haidian district were gathered by using the Location Based Service open platform of Amap [25]. We then included all supermarket chains with more than four markets in our study. Besides, we reviewed the Grocery Market Share data from Kantar Worldpanel research [26] to ensure that we included all supermarkets in the top 10 Grocery Market Share of China in this district. One supermarket was selected for each supermarket chain.

A total of 15 supermarkets were included in this study and they were Wu Mart, Yonghui Superstores, Wal-Mart, Cuiwei, Merry Mart, Carrefour, Xingfu, Hua Lian, Century Mart, Chaoshifa, Jingkelong, Vanguard, Auchan, Shijijiajia, and Shijihualian.

### Definition and selection of NCSSBs

The categories of NCSSBs included juice drinks, tea-based beverages, sports drinks, and energy drinks [10], whose definitions were listed in Additional file 1**: Table S1** for comparison with other studies [7, 27].

### Data collection

The data were collected from product packaging and nutrient information panels of each NCSSBs by a snapshot in time. For each NCSSBs, its company name, product name, serving size, nutrient content of carbohydrate, sugar and energy per 100 ml were extracted. The information of country of origin on the package of products and nutrition labels were used to determine if the NCSSBs were local or imported. For those NCSSBs without sugar content information on their nutrition labels, we used carbohydrate content as a replacement. NCSSBs without the information (carbohydrate/sugar/energy) we needed on their nutrition labels were not investigated in this study. When any product which had been photographed in a previous supermarket showed in subsequent supermarkets, it would not be photographed again. Some brands sold the same formulation in different serving sizes, and we only included one product of one formulation.

### The recommendations for free sugar intakes

Two different recommendations for free sugar intakes were used in our study to evaluate sugar content in different categories of NCSSBs. The Food Standards Agency in the UK released a guideline on front of pack color-coded labelling for drinks [28]. Color coding was used to show if the sugar content in a product was high or not, which was based on sugar content on the nutrition label using the following criteria (sugars—red/high> 13.5 g/portion or > 11.25 g/100 mL, amber/medium> 2.5 and ≤11.25/100 mL, green/low ≤2.5 g/100 mL). The World health Organization (WHO) recommended that a restriction of 25 g daily free sugar intake would provide additional health benefits in reducing non-communicable diseases [1].

### Statistical analysis

Epidata was used for data entry and all data were double checked by the authors (Jin and Peng). Sugar and energy content in different NCSSBs were presented as median and interquartile ranges (IQR). The nonparametric Kruskal-Wallis test was used to determine differences across four NCSSBs categories and between imported and local products. Products that meet the recommendations for free sugar intakes were presented as number and proportion. McNemar–Bowker test was used for comparison of the proportion of products with ‘Red’ label between per 100 ml criterion and per serving criterion according to the UK front of pack color-coded labelling. For NCSSBs with sugar and carbohydrate contents information on their nutrition labels, Spearman’s rank correlation coefficient and related-samples Wilcoxon signed-rank test were used to explore the relationship between two contents. Sensitivity analysis was performed to compare the results between NSCCBs with and without sugar information. We tested whether the replacement of sugar content with carbohydrate content in NCSSBs induced bias or not. Statistical analyses were conducted using SAS, version 9.4, and a *P* value< 0.05 was considered statistically significant.

## Results

A total of 463 NCSSBs met the inclusion criteria and were included in our analysis. Of the 463 products, 323 were juice drinks, 96 were tea-based beverages, 32 were sports drinks and 12 were energy drinks.

**Fig. 1:**
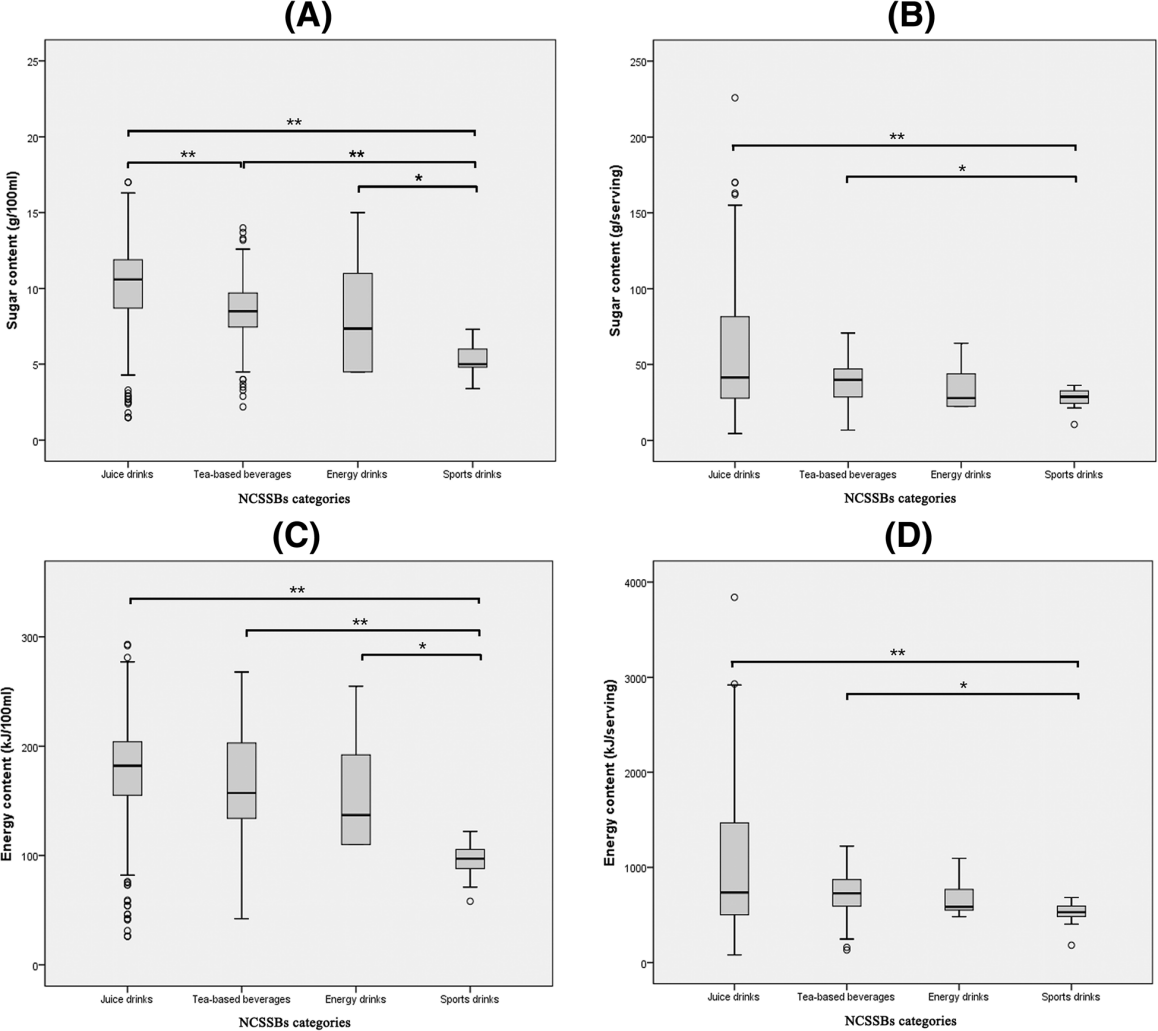
Multiple comparisons of sugar and energy content among different NCSSBs categories. (A) Sugar content in different NCSSBs categories (g/100 ml). (B) Sugar content in different NCSSBs categories (g/serving). (C) Energy content in different NCSSBs categories (kJ/100 ml). (D) Energy content in different NCSSBs categories (kJ/serving). *:*P* < 0.05. **:*P* < 0.01. NCSSBs, non-carbonated sugar-sweetened beverages

Table 1 showed sugar and energy content in different NCSSBs. In general, the median of serving size was 500 (IQR: 310–600) ml. The median of sugar content was 9.6 (IQR: 7.1–11.3) g/100 ml or 39.0 (IQR: 27.5–53.3) g/serving. The median of energy content was 176 (IQR: 121–201) kJ/100 ml or 703 (IQR: 502–980) kJ/serving. There were statistical differences in sugar and energy content among different categories of NCSSBs (both per 100 ml and per serving). Multiple comparison tests revealed juice drinks had higher sugar content per 100 ml than tea-based beverages, and sports drinks, and sports drinks had the lowest sugar and energy content per 100 ml (data were shown in Fig. 1). Besides, compared with local products, imported products had higher sugar content (median: 11.2 g/100 ml vs 9.6 g/100 ml, *P* < 0.05) and energy content (median: 193 kJ/100 ml vs 166 kJ/100 ml, *P* < 0.05).

**Table 1:** Sugar and energy content in different NCSSBs

Categories	N	Serving size (ml) Median (Range)	Sugar content (g) Median (Range)		Energy content (kJ) Median (Range)	
			Per 100 ml	Per serving	Per 100ml	Per serving
Total	463	500 (310–600)	9.6 (7.1–11.3)	39.0 (27.5–53.3)	176 (121–201)	703 (502–980)
Juice drinks	323	488 (260–1000)	10.4 (8.5–11.7)	41.6 (27.8–82.0)	180 (155–205)	737 (498–1490)
Tea-based beverages	96	500 (450–500)	8.5 (7.5–9.7)	40.0 (28.7–47.0)	159 (134–205)	737 (603–892)
Sports drinks	32	580 (500–600)	5.0 (4.8–6.0)	28.8 (24.5–32.8)	96 (88–105)	527 (481–594)
Energy drinks	12	500 (400–500)	7.4 (4.5–11.0)	28.0 (22.5–44.0)	138 (109–193)	586 (548–770)
*P*		0.01	< 0.01	< 0.01	< 0.01	< 0.01

*Abbreviation: NCSSBs*, non-carbonated sugar-sweetened beverages

Table 2 showed the sugar content in different categories of NCSSBs according to different recommendations for free sugar intake. Based on the UK recommendation, 117 (25.3%) products of NCSSBs received a ‘red’ color-coded label in per 100 ml criterion, while 441 (95.2%) products of NCSSBs labelled ‘red’ in per portion criterion. Meanwhile, 378 (81.6%) products had sugar content exceeding the WHO daily free sugar intake recommendation. In all NCSSBs, the proportion of products receiving ‘red’ label was significantly different when using per 100 ml criterion and per portion criterion (McNemar–Bowker test, χ^2^ = 7.810, *P* < 0.05). Juice drinks had the highest percentage of products (32.2%) exceeding the UK per 100 ml criterion while tea-based beverages had the highest percentage of products (83.3%) exceeding the WHO criterion. All energy drinks received ‘red’ label according to the UK per serving criterion.

**Table 2:** Description of sugar content in different NCSSBs according to the UK criterion for sugar intake and WHO recommendation

Categories	Number (%) of products with ‘Red’ label according to the UK front of pack color-coded labelling		Number (%) of products with free sugar>WHO recommendation^β^	Number (%) of products with sugar labelling
	> 11.25 g/100 ml	> 13.5 g/portion^α^		
Total	117 (25.3)	441 (95.2)	378 (81.6)	82 (17.7)
Juice drinks	104 (32.2)	304 (94.1)	268 (83.0)	63 (19.5)
Tea-based beverages	11 (11.5)	94 (97.9)	80 (83.3)	10 (10.4)
Sports drinks	0 (0)	31 (96.9)	23 (71.9)	9 (28.1)
Energy drinks	2 (16.7)	12 (100)	7 (58.3)	0 (0.0)

^α^:The serving criterion of UK guidance of front of pack color-coded labelling for drinks was> 13.5 g/portion if serving size> 150 ml

^β^: The recommendation for daily free sugar intakes from the WHO was 25 g

*Abbreviation*: *NCSSBs*, non-carbonated sugar-sweetened beverages

Among 463 NCSSBs, 82 (17.7%) products had sugar content on the nutrition labels. Juice drinks had highest proportion of products with sugar labelling (19.5%) while none of the energy drinks had sugar content on the label (shown in Table 2). The percentage of imported products with sugar content information was statistically higher than local products (26.0% vs 12.2%, χ^2^ = 14.540, *P* < 0.01). In those 82 NCSSBs with sugar content information, the Spearman’s rank correlation coefficient between carbohydrate and sugar content was 0.899 (*P* < 0.0001). The paired differences between sugar and carbohydrate contents was 0.76 ± 0.16 g/ 100 ml (shown in Additional file 2**: Table S2**).

The sensitivity analyses showed that the sugar content in NCSSBs with both sugar and carbohydrate information was insignificantly different from those without sugar information, which we used carbohydrate content as alternatives (9.4 g/100 ml vs 9.7 g/100 ml, 39.0 g/serving vs 39.0 g/serving). Similar results were found in the proportion of products exceeding recommendation levels between NCSSBs with and without sugar information (shown in Additional file 3**: Table S3**).

## Discussion

To our knowledge, this is the first cross-sectional survey to investigate sugar and energy content in NCSSBs in the Chinese supermarkets. In our study, we found high sugar and energy content in all four NCSSBs categories, and few of them had sugar content information on their nutrition labels especially in local products. When considering the serving size, free sugar in most NCSSBs exceeded the recommendations of free sugar intakes from the UK and WHO.

In general, our study confirmed that the sugar and energy content of NCSSBs were remarkably high in China, which were similar to those of CSSBs in developed countries. The average free sugars content in CSSBs was 30.1 ± 10.7 g/330 ml in the UK [6]. The mean total sugar of pop/soda and iced teas with added sugar was 10.6 ± 5.0 g/100 ml in Canada [8]. Our results confirmed that NCSSBs were not the best alternatives to CSSBs because of their high free sugar content, and this misconception should be communicated to the consumers. Government should take steps to reduce the sugar content in NCSSBs, especially paying attention to imported products because of the higher sugar content in them than local products.

Given the adverse health effects of NCSSBs, campaigns to restrict the consumptions of NCSSBs have been launched in several countries. In the USA, a campaign of eliminating fruit juice to reduce childhood obesity has started [29], and a bill that would ban the sale of energy drinks to minors has also been introduced [30]. In the UK, many supermarkets have banned the sale of caffeinated energy drinks to under-16 s [31]. Considering the rapidly expanding market of NCSSBs in China, the hazard of NCSSBs must be highlighted and measures should be taken.

Attention should also be paid on consumers’ low awareness of the sugar content in NCSSBs, which may be partly explained by the mass exposure to advertising. For example, in a national survey conducted in the UK, consumers showed serious misconception of the sugar content in juice drinks with 48% underestimation [32]. Another study also found that consumers’ awareness of health risks and sugar content of SSBs and fruit juice was low [33]. Therefore, higher-quality, rigorous interventions on consumers’ media literacy are needed to enhance consumers’ awareness towards the adverse health effects of NCSSBs and government should create strong policies to promote consumption of healthy drinks.

With the varied serving size of NCSSBs in China, it’s of importance to reduce and restrict the serving size in SSBs including NCSSBs. Serving size of a product is an important determinant of sugar content per serving. Consumers tended to drink more when the serving size was larger [34], leading to extra energy intakes [35]. Studies have confirmed the positive association between portion size and excess weight gain in Brazil [36] and the USA [37]. In Australia, predicted model suggested that a restriction of serving size to 375 mL on packaged single-serve SSBs resulted in lower body weight and less economic burden [38]. In some states of the USA, several policies have been released on restricting the serving size of SSBs [39].

Improving the standard of nutrition label in China is of great importance. Nutrition labels on pre-packaged foods are among the most accessible sources of nutrition information, which are perceived as a highly credible source of information and are used by many consumers to guide their selection of food products [21]. In our study, however, only a few products (17.7%) had sugar content on their nutrition labels because sugar content information is not mandatory in China. 60.2% of them were imported products, which indicated that foreign beverage companies may pay more attention to sugar labelling because of the compulsory regulation in those countries (for example, the UK [28] and the USA [40]). Of note, none of the energy drinks were with sugar labelling while all of them had high sugar content. Given that sugar content was considered as a primary concern when evaluating the nutritional quality of soft drinks [9], the lack of sugar content information is more likely to mislead consumers into focusing on the so-called health effects of NCSSBs. Government needs to mandate sugar labelling and improve the intelligibility of the nutrition information, which could be helpful for consumers to better use the nutrition labels for selecting beverage.

Several limitations should be noted. First, this cross-sectional study was only conducted in one district of Beijing, but we included all supermarket chains with more than four markets and all supermarkets in the top 10 Grocery Market Share of China in this district. Thus, it’s reasonable to assume the NCSSBs products included in our study are similar to those products sold in other districts in Beijing and most urban areas in China. Second, we used carbohydrate content to substitute sugar content in those NCSSBs without sugar information. However, sensitivity analyses showed similar results. Further study is needed when sugar content information becomes mandatory in China. Third, NCSSBs without the information we needed on their nutrition labels were not investigated in this study. Further study could examine the sugar profile in these products using assay methods.

## Conclusions

The sugar and energy content of NCSSBs were high in China and few of the NCSSBs had sugar content information on their nutrition labels especially in local products. Furthermore, when considering the serving size, the free sugar in most NCSSBs exceeded the recommended level according to the UK and the WHO criteria. Measures including developing better regulation of labelling, reducing sugar content and restricting the serving size are needed for reducing sugar intakes in China.

## Additional files

Additional file 1: **Table S1.** The definitions of juice drinks, tea-based beverages, sports drinks and energy drinks. (DOCX 14 kb)

Additional file 2: **Table S2.** Sugar content information in different categories of NCSSBs. *: *P* < 0.05. Abbreviation: NCSSBs, non-carbonated sugar-sweetened beverages. (DOCX 14 kb)

Additional file 3: **Table S3.** Comparison between NCSSB with and without sugar information according to different recommendations. ^α^:The serving criterion of UK guidance of front of pack color-coded labelling for drinks was> 13.5 g/portion if serving size> 150 ml. ^β^: The recommendation for daily free sugar intakes from the WHO was 25 g. Abbreviation: NCSSBs, non-carbonated sugar-sweetened beverages. (DOCX 14 kb)

## Data Availability

The datasets used and/or analysed during the current study are available from the corresponding author on reasonable request.

## Abbreviations

CSSBs: carbonated sugar-sweetened beverages
IQR: interquartile ranges
NCSSBs: non-carbonated sugar-sweetened beverages
RTD: ready-to-drink
SSBs: sugar-sweetened beverages
WHO: World Health Organization
