# Supplementary material for: The sugar and energy in non-carbonated sugar-sweetened beverages: a cross-sectional study
Source: BMC Public Health. 2019 Aug 20;19:1141. doi: 10.1186/s12889-019-7486-6 (PMC6700807; doi:10.1186/s12889-019-7486-6)
Supplement: Supplementary file 1 — Table S1. The definitions of juice drinks, tea-based beverages, sports drinks and energy drinks. (DOCX 14 kb) [file 12889_2019_7486_MOESM1_ESM.docx]

**Additional file 1: Table S1 The definitions of juice drinks, tea-based beverages, sports drinks and energy drinks**

| Categories | Definitions |
| --- | --- |
| Juice drinks | Beverages containing 1% to 99% juice. |
| Tea-based beverages | Ready-to drink tea (sweetened or unsweetened, flavored or unflavored). |
| Sports drinks | Functional drinks specifically designed to help athletes and other active people hydrate before, during and after exercise. |
| Energy drinks | Energy drinks provide functional benefits by boosting energy and alertness. The functionality is obtained from ingredients such as glucose, caffeine or taurine. |
