# Supplementary material for: The sugar and energy in non-carbonated sugar-sweetened beverages: a cross-sectional study
Source: BMC Public Health. 2019 Aug 20;19:1141. doi: 10.1186/s12889-019-7486-6 (PMC6700807; doi:10.1186/s12889-019-7486-6)
Supplement: Supplementary file 2 — Table S2. Sugar content information in different categories of NCSSBs. *: P < 0.05. Abbreviation: NCSSBs, non-carbonated sugar-sweetened beverages. (DOCX 14 kb) [file 12889_2019_7486_MOESM2_ESM.docx]

**Additional file 2: Table S2 Sugar content information in different categories of NCSSBs**

| Categories | Spearman’s rank correlation coefficient between carbohydrate and sugar content | Paired differences between carbohydrate and sugar content[mean±SD (g/100ml) ] |
| --- | --- | --- |
| Total | 0.899* | 0.76±0.16* |
| Juice drinks | 0.801* | 0.96±0.20* |
| Tea-based beverages | 0.994* | 0.15±0.04* |
| Sports drinks | 1.000* | 0 |
| Energy drinks | - | - |

*: *P*<0.05.

Abbreviation: NCSSBs, non-carbonated sugar-sweetened beverages.
