# Supplementary material for: The sugar and energy in non-carbonated sugar-sweetened beverages: a cross-sectional study
Source: BMC Public Health. 2019 Aug 20;19:1141. doi: 10.1186/s12889-019-7486-6 (PMC6700807; doi:10.1186/s12889-019-7486-6)
Supplement: Supplementary file 3 — Table S3. Comparison between NCSSB with and without sugar information according to different recommendations. α:The serving criterion of UK guidance of front of pack color-coded labelling for drinks was> 13.5 g/portion if serving size> 150 ml. β: The recommendation for daily free sugar intakes from the WHO was 25 g. Abbreviation: NCSSBs, non-carbonated sugar-sweetened beverages. (DOCX 14 kb) [file 12889_2019_7486_MOESM3_ESM.docx]

**Additional file 3: Table S13Comparison between NCSSBs with and without sugar information according to different recommendations**

|  | NCSSBs with sugar information  N=82 | NCSSBs without sugar information  N=381 | *P* |
| --- | --- | --- | --- |
| Number (%) of products with ‘Red’ label according to the UK front of pack color-coded (>11.25g/100ml) | 17 (20.7) | 100 (26.2) | 0.184 |
| Number (%) of products with ‘Red’ label according to the UK front of pack color-coded (>13.5g/portion)^α^ | 80 (97.6) | 361 (94.8) | 0.219 |
| Number (%) of products with free sugar>WHO recommendation^β^ | 64 (78.0) | 314 (82.4) | 0.218 |

^α^:The serving criterion of UK guidance of front of pack color-coded labelling for drinks was>13.5g/portion if serving size>150ml.

^β^: The recommendation for daily free sugar intakes from the WHO was 25g.

Abbreviation: NCSSBs, non-carbonated sugar-sweetened beverages.
